# Pursuing a hierarchical taxonomy in a water-cooled data center room

**DOI:** 10.1038/s41598-026-68222-1

**Published:** 2026-08-24

**Authors:** Vlatko Milić, Patrik Thollander, Maria Andersson, Jim Enkel, Bahram Moshfegh

**Affiliations:** 1 Division of Energy Systems, Department of Management and Engineering, Linköping University, 581 83 Linköping, Sweden; 2 Division of Building, Energy and Environment Technology, Department of Technology and Environment, University of Gävle, 801 76 Gävle, Sweden; 3 Linköping Lab Operations, Ericsson AB, 581 12 Linköping, Sweden

**Keywords:** Taxonomy, Data center, Detached in-rack water cooling system, Energy end-use, Energy performance indicators, Energy infrastructure, Information technology

## Abstract

Despite the growing number of scientific investigations on developing taxonomies in various industries and categorizing energy end-use (EEU) processes, research on such frameworks in the data center (DC) sector remains limited, restricting systematic analysis of energy use, benchmarking, and identification of energy efficiency measures. This study seeks to achieve two key objectives: (1) to create a hierarchical taxonomy for categorizing EEU process, (2) and to identify the most relevant energy performance indicators (EnPIs). The methodology combines hierarchical classification of energy flows with quantitative analysis based on time-resolved operational data, allowing for detailed analysis of dynamic system behaviour and allocation of energy use across processes, applied to a DC room with a detached in-rack water cooling system in Sweden operated by Ericsson. The results of this paper present a novel hierarchical taxonomy for a water-cooled DC room with six defined EEU processes, of which four are related to data processing processes and two to support processes. Notably, over 99.9% of total EEU is connected to data processing processes which is a significantly higher figure compared to other production processes in other sectors. Moreover, the established EnPIs can facilitate benchmarking between DCs with similar infrastructures and for monitoring the energy use of various EEU processes. The proposed methodology is transferable to other DC environments with comparable system boundaries and data availability, and its reproducibility is supported by the structured and data-driven methodology, which enables replication of the analytical approach given comparable data availability.

## Introduction

### Background

Without action the impacts of climate change will be immense on our world in terms of more frequent heat waves, rising sea levels, threats to water supply etc. More than 10 million people die annually from air pollution as a consequence of combustion of fossil fuels^1^. To address the climate crisis, including other topical issues for humanity such as peace and poverty, Agenda 2030 has been formed by the United Nations, which is the most ambitious strategy ever to achieve sustainable development globally^2^. An important part in Agenda 2030 is the development of resilient infrastructure and sustainable industrialization, including significantly increase the access to information and communications technologies. The transformation to a digital society is one of the foundations to accelerate energy-efficient technologies. In fact, the central role of digitalization for fulfilling sustainability goals is highlighted in the European Commission’s digital compass established in 2021^3^. Nonetheless, despite the large opportunities with digital technologies, as demonstrated by major companies such as Facebook and Google, along with their reliance on data centers (DCs)^4^, challenges exist to ensure a sustainable digitalization. The amount of data globally is increasing exponentially with associated impacts on energy use and resource exploitation from DCs. According to the International Energy Agency (IEA)^5^, the total electricity usage of DCs worldwide was 415 TWh in 2024 corresponding to around 1.5% of the global electricity usage^5^. Interestingly, global electricity consumption in DCs has increased by approximately 12% per year since 2017, exceeding the growth rate of total electricity consumption by more than a factor of four. By 2030, DC electricity use is projected to more than double, reaching approximately 945 TWh. The exponential growth in electricity demand indicates that energy efficiency in DCs is an important step to achieve a sustainable development. Additionally, energy efficiency in DCs allows for increased profitability by decreasing power bills. This will most likely have an even more prominent role in future with increasing electricity prices.

In order to mitigate climate change, the EU has established the European Green Deal with the objective of making Europe the first climate neutral continent^6^. A total of 1.8 trillion euro will be invested within the union to fulfill the set goals. An important key in the work with the European Green Deal is the necessity of redirecting investments towards sustainable projects to build societies that are resilient against climate change^7^. Moreover, by providing structured frameworks for organizing complex information, the concept of taxonomy serves as an important method for fostering sustainability. The term “taxonomy” can be referred to the “theory and practice of delimiting and classifying different kinds”^8^. The investigation performed by Söderström^9^ in 1996 is one of the earliest papers published in the field of taxonomies targeting categorization of industrial EEU. The research was characterized by the use of unit processes, i.e., dividing all processes into either production processes or support processes. The production processes are connected to processes used to produce products while support processes are activities that do not directly contribute to production but provide support for it.

The modern categorization of plants and insects emerged in the sixteenth century by Carl Linnaeus and is still today standard practice. A corresponding categorization of industrial energy end-use (EEU) processes is substantially less complex, but there is still a general lack of knowledge in the field. To date, a number of scientific investigations have been performed targeting EEU categorization in industry, such as in the wood, food and metal industry^10–12^. However, to the author’s knowledge, research on developing taxonomies for EEU in DCs is a neglected field of research. Consequently, there is a need to broaden the research on categorizing the EEU processes in industry by also investigating DCs. This is especially of interest considering the ongoing digitalization and increase in the amount of data globally.

### Objective and novelty of the research

So far researchers have categorized EEU processes and presented taxonomies in various types of industries. However, to the best of the authors’ knowledge, research on categorizing EEU processes in DCs is scarce. The need for a common taxonomy in DCs is emphasized with the exponential growth in data volume globally. This research aims to (1) present a hierarchical taxonomy for a DC room to categorize EEU processes with hierarchical levels and (2) to identify suitable energy performance indicators (EnPIs).

Classification of EEU processes consisting of both energy use for cooling and electricity in DCs is novel in nature. The development of an architecture database will aid in increasing the knowledge of EEU processes in DCs, as well as to enable a comparison between these. The taxonomy is based on a DC room with detached in-rack water cooling system situated in Linköping, Sweden. Moreover, this research makes an important contribution to analyze and structure EEU processes in DCs. The results are relevant for policymakers, authorities, DC industries and the research community in the pursuit of classifying EEU processes in DCs, and designing database architectures related to the most energy-intensive EEU processes.

## Theoretical background

### Taxonomies in research

Taxonomies on categorizing industrial EEU has been used in numerous scientific papers in various contexts. For example, Sommarin et al.^13^ developed a taxonomy for the foundry industry, Blomqvist and Thollander used the taxonomy to create a unique architecture of real energy efficiency measures^14^, Andersson et al.^15^ developed a framework enabling benchmarking and KPI development in the pulp and paper industry, and Johnsson et al.^12^ developed a taxonomy for the wood industry and thanks to that, were able to categorize the Swedish wood industry’s total energy balance from supply of energy down to end-users. Furthermore, Kanchiralla et al.^10,16^ developed taxonomies for the food and mechanical engineering industry respectively, and Haraldsson et al.^11^ have developed a taxonomy for the aluminum industry.

### DCs

The term DC can be interpreted differently by different persons and actors^17^. Other common terms for a DC include, among others, data hall, data warehouse and high-performance lab. The common denominator of DCs is to process information. According to the U.S. Environment Protection Agency, a DC is characterized by:

“*Primarily electronic equipment used for data processing (servers), data storage (storage equipment), and communications (network equipment). Collectively, this equipment processes, stores, and transmits digital information*.”

"*Specialized power conversion and backup equipment to maintain reliable, high-quality power, as well as environmental control equipment to maintain the proper temperature and humidity for the ICT equipment*.”^17^

A DC commonly consists of different rooms with different designs. Each room consists of rows of cabinets, or racks, which contain server units of hardware. A rack can hold IT equipment, such as servers or storage devices, each of which can be of a single or multiple unit size, or even custom equipment, depending on the specific application within the DC. The infrastructure of a DC room also includes cooling devices, lighting and other equipment.

A consequence of the ongoing digitalization in society is growing energy use in DCs. There is an exponential growth in demand for data services globally^18^ which will consequently affect the energy usage and resource utilization from this sector. However, it is important to be aware that the performance per watt of servers in is increasing, which makes predictions of future energy use from DCs a difficult task. It is important to map the use of different energy carriers in DCs to obtain a holistic perspective on the future consequences in energy systems. This may aid in the development of resilient energy systems which contribute to national energy security.

To handle the increasing energy use in DCs, the EU Code of Conduct on Data Centre Energy Efficiency has been established by the Joint Research Center (DG JRC) from the European Commission^19^. The overall aim is to reduce energy use in a cost-effective manner whilst maintaining a DC’s critical functions. Academics, operators, consultants and national bodies are examples of experts who have contributed to the development of the code of conduct. Among the key parts of the code of conduct include auditing of the current equipment and their delivery output, as well as increasing efficiency in cooling systems. These actions are facilitated by a knowledge base related to EEU processes in DCs, which is highlighted in this research.

### EnPIs

As highlighted in the standard ISO 50,006 (Energy management systems—Measuring energy performance using energy baselines (EnB) and energy performance indicators (EnPI)—General principles and guidance), it is necessary to have knowledge on the dynamic energy use of various parts in organizations, such as facilities, systems, processes and equipment, to successfully manage energy performance^20^. This is made possible by the implementation of EnPIs which are defined as “*quantitative value or measure of energy performance defined by the organization*” by ISO 50,006^20^. Common units for describing EnPIs include energy use (kWh), specific energy use per produced unit (kWh/unit) and peak power. In order to allow for comparison of EnPIs over time, energy baselines (EnBs) are used. EnBs are reference values describing the energy performance which also allow for determining changes in EnPIs. Consequently, this facilitates decisions related to improvement measures for increased energy efficiency.

EnPIs can be divided into three different boundary levels according to ISO 50,006^20^: organizational, system and individual level, also commonly titled process level, according to Table 1. Examples of studies using these boundary levels include^16,21^ addressing the Swedish engineering industry and the Swedish paper and pulp industry, respectively. Moreover, a study by Sommarin et al.^13^ divided the EnPI boundary into three parts: overall figures, production-process and support-process. This shows possibilities for using different boundary levels while developing EnPIs. Nonetheless, it is important to be aware of that production processes vary significantly between industrial sectors^16^. Hence, it is important to develop EnPIs specifically for the DC industry, especially when considering its unique nature compared to other industries. Moreover, the knowledge gap for how EnPIs are investigated in the scientific community and what the needs are in industry has been highlighted by May et al.^22^. The identified knowledge gaps include e.g., lack of guidelines for selecting EnPIs, inadequate technology monitoring and lack of tools to be used in the development of EnPIs. The study by Bunse et al.^23^ is in line with the findings by May et al.^22^, which stresses the general need for EnPIs in industry.

**Table 1:** Summary of EnPI boundary levels according to ISO 50,006^20^.

EnPI boundary level	Short description
Individual equipment/process/facility	Boundary level around an equipment, process or facility
System	Boundary level around a number of interacting equipment, processes or facilities
Organizational	Boundary level around a number of equipment, processes or facilities while considering the energy management by the organization

From a DC perspective, the Power Usage Effectiveness (PUE) has become an industry standard for measuring energy performance^24^. In fact, Horner and Azevedo^24^ mentions that the quest for an optimal PUE has sparked a competition between operators of DCs. PUE describes the power required to operate and cool the DC in relation to the power needed for the operation of the IT equipment, e.g., server units, as shown in Eq. (1). Lower PUE values are therefore better, with 1.0 being the optimum. If all power supplied to the DC is used by the IT equipment, the PUE would be 1.0.

1 $$PUE=\frac{Total facility power}{IT equipment power}$$

Despite that the PUE value is useful for describing the energy efficiency of a DC, it does not alone provide a complete understanding of the performance of a complex system as a DC^24^. For example, the ambient climate is not considered in the PUE value. In addition, another limitation with the use of PUE is that IT equipment efficiency is overlooked for describing the energy efficiency of a DC^24^. In practice, this means that a DC facility with old inefficient servers could have a better PUE than a DC facility with new IT equipment. In order to generate a more comprehensive picture of the energy efficiency of DCs, the DC sector is developing complementary EnPIs to supplement the PUE value. A popular and increasingly more important measure in DCs is floating-point computing power, which is expressed as Flops/W^25^. In 2013, the first ranking of computers based on their floating-point computing power was introduced in the Green 500 list^26^. The use of Flops/W allows for assessing the amount of computation that can be performed for each watt of electricity used.

### Cooling techniques

The EU Code of Conduct on Data Centre Energy Efficiency highlights that cooling procedures in DCs represent a significant potential for energy efficiency^19^. This also accords with the study by Moazamigoodarzi et al.^27^ who points out the high cooling demand in DCs, as well as opportunities for energy savings due to mixing of hot and cold air. Traditionally, DCs have row-based design of IT power loads which means that there are hot and cold aisles^17^. Rows with server rack fronts are called cold aisles, and rows where the heated return air exits the equipment (backs of servers) are typically called hot aisles. Historically, air cooling has been the most common cooling technique in DCs^28^. Below follows a summary of common cooling techniques used in DCs.

Air cooling is the most common used cooling technique in DCs despite a lower heat transfer capability compared to liquids^27^. The reason for this is the ease of application and operation. Server racks are in general arranged into cold and hot aisles in air-cooled DCs^29^. The cold air generated by the Computer Room Air Handler (CRAH) unit is directed to cold aisles through the floor or ceiling. It is also possible to drive the cold air horizontally via CRAH units located in connection with the cold aisles. The heat generated in the server units is returned to the hot aisles. With the increase in power density of DCs in recent times, problems with insufficient cooling of CRAH units have emerged^29^. Therefore, to deal with the issue of inadequate cooling supply in DC, new cooling techniques have been introduced^30^. Liquid cooling-based systems have gained interest in the industry to improve cooling strategies, and to achieve energy savings related to cooling as highlighted by Greenberg et. al^31^. Liquid cooling-based systems use water or a dielectric liquid to transfer away heat from server units^32^. There are different types of liquid cooling-based systems, and the key distinction between them is how close the liquid is to the server equipment—whether the cooling liquid is in direct contact with the components, as in direct liquid cooling, or nearby without direct contact, as in indirect liquid cooling. For example, as shown by Li et al.^33^ there are possibilities for directly cooling servers in liquid cooling tanks as a means of dissipating heat. In this approach, maintaining good temperature uniformity is crucial to prevent server malfunctions. The recent review on direct liquid cooling methods by Kong et al.^34^ outlines various possibilities for cooling DCs, including spray cooling, where high-speed jets are concentrated in a limited space, creating a very thin boundary layer that enhances heat transfer efficiency. Another method discussed is direct microchannel cooling, where coolant flows through manifold microscale channels within or close to the chip, removing heat near the heat-generating region with high heat transfer efficiency. However, as the review states, direct liquid cooling methods often present challenges related to integration complexities. In this regard, air to liquid cooling is simple to implement but faces constraints for high-power servers^35^.

It is important to be aware that the selection of cooling strategy is a multifaceted task. Other factors than energy density that affects the choice of cooling strategy includes, among others, local climate and local fuel prices for energy carriers^36^. Another influencing factor includes possibilities for connection to the surrounding energy system for the use of district cooling in the DC. District cooling provides cooled water from a centralized cooling plant through a pipe distribution network. Free cooling uses ambient air for cooling buildings either directly or with an intermediate energy carrier such as water. An essential aspect of free cooling is the monitoring of ambient climate to ensure appropriate temperature and humidity levels^37^. The key advantage with free cooling is the low amount of electricity required in the cooling process. Compressor cooling systems are unique in their ability to generate cooling whenever needed and are not reliant on external factors. The dependence of external factors is a disadvantage with district cooling and free cooling, which require a surrounding infrastructure and cold outdoor temperatures, respectively.

## Description of the methodology

**Fig. 1:**
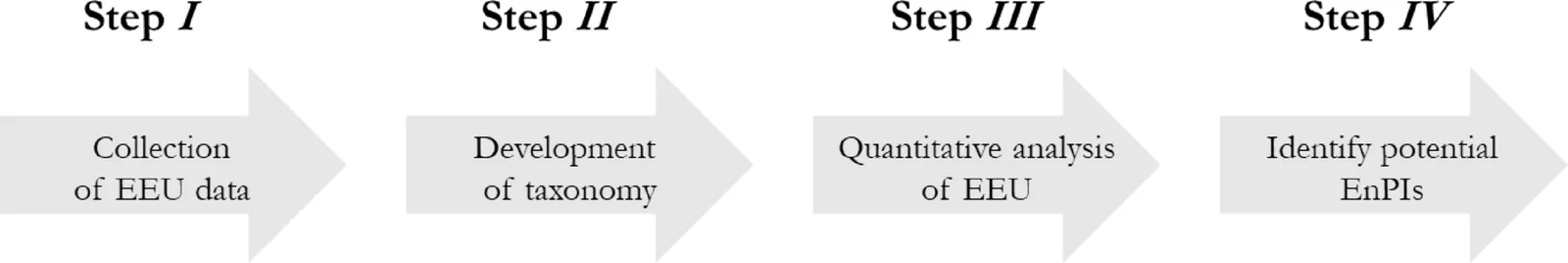
Schematic of the proposed methodology for the development of a taxonomy and identifying EnPIs.

The methodology applied in this research is inspired by Kanchiralla et al.^16^ and can be divided in four main steps according to Fig. 1. A combination of an axiomatic approach, i.e., a hierarchical classification consisting of multiple layers comparable with biological taxonomy, and quantitative analysis is used. In Step *I*, EEU data are collected. Step* II* consists of the development of a taxonomy, which is based on hierarchical classification of the energy use data. Step *III* consists of a quantitative approach for analyzing EEU. In Step *IV*, the objective is to identify potential EnPIs by investigating relevant parameters connected to energy performance.

### Collection of EEU data

Digital technologies are a key instrument to collect and analyze data in a time-effective manner^38^. In addition, by using sensors for measurements and thereafter collecting digital data it is possible to generate accurate and detailed information about EEU processes with different time resolutions.

In this research, EEU data has been collected from existing building management systems and the Supervisory Control and Data Acquisition (SCADA) system, constantly monitoring and logging energy use and environmental parameters. Collected variables include energy use needed for server units, cooling of the server units, and total electricity use of the DC room. The time resolution of the data is five minutes. To the best of the authors’ knowledge, such datasets for generating database architectures in DCs, are unique in scientific studies based on the non-systematic literature search performed in this research.

### Development of taxonomy

The scope of this research is a DC room operated by the global telecommunications company Ericsson. The processes in the investigated DC room were divided into either data processing processes or support processes. Data processing processes are connected to the process of transforming the input resources to an economical output, which are commonly labeled as production processes in other sectors as mentioned in Section “EnPIs”. In the case of the DC industry, this includes the operation of server units that are used for e.g., processing and storage of data. Moreover, the support processes, i.e., processes supporting the data processing processes, were defined with inspiration from the taxonomy developed by Söderström^9^, while the support processes identified for the studied DC room are presented in Section “Taxonomy of EUU for a DC room”. To identify relevant support processes, observations from on-site visits were used. These were validated through communication with Ericsson.

The identification and classification of EEU processes were based on a combination of criteria. First, processes were categorized according to their functional role, distinguishing between data processing processes and support processes. Second, the inclusion of processes was guided by on-site observations and system understanding of the studied DC room, Third, data availability influenced the level of detail at which processes could be represented. Finally, the classification was iteratively refined and validated through workshops, ensuring consistency and relevance for the specific DC environment.

### Quantitative analysis of EEU

The quantitative analysis of EEU data is performed using energy data collected from the building management systems. The calculations of EEU on an aggregated level was conducted using a bottom-up approach, which was inspired by Yin^39^. As mentioned by^10,40^, this approach enables detailed analysis of production processes, which are referred to data processing processes in this research due to the unique character of DCs. The EEU on aggregated levels is calculated by aggregating EEU from lower levels. The aggregation of EEU data is based on the bottommost level obtained from the data collection, referring to the most detailed available measurement level.

The power associated with ventilation can be quantified according to the European standard EN 16,798–3:2017^41^, as presented in Eq. (2).

2 $$SFP=\frac{{P}_{fan}}{q}$$

where SFP is the specific fan power (W/(m^3^/s)), *P*_fan_ (W) corresponds to the electrical power used to operate the ventilation fan, and *q* is the total airflow rate through the ventilation fan (m^3^/s). In combination with the total airflow rate and operational hours, the SFP value enables the quantification of total energy use for ventilation. Moreover, the energy use for pumping the cooling water (*E*_pumping_) is calculated utilizing average power (*P*_pumping_) together with the operational hours (*h*), as shown in Eq. (3).

3 $${E}_{pumping}={P}_{pumping} \cdot h$$

**Fig. 2:**
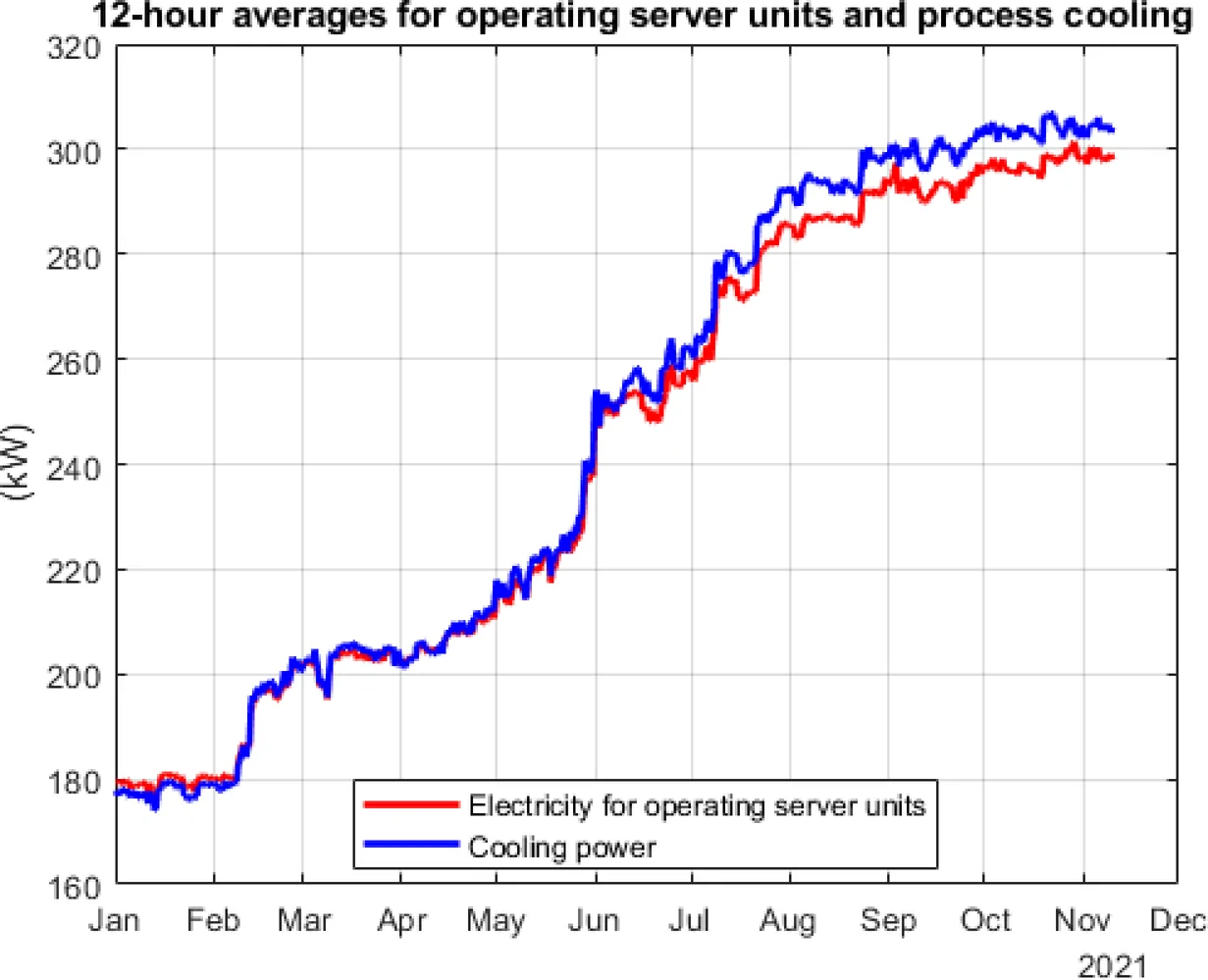
Electricity usage for operating server units and process cooling (kW).

The energy use for the investigated DC room can also be determined by using a top-down approach. This is achieved by summing up the energy use for all energy carriers. Consequently, the accuracy of the results can be ensured by using both a top-down and bottom-up approach. As in the case for EEU data, information about the energy use from energy carriers was collected from building management systems and facility management consultants. Figure 2 displays data related to the electricity use for operation of server units and process cooling, using a 12-h time step. The power required for operating server units and for process cooling increases by around two-thirds over the investigated time period, driven by the installation of 1,400 server units and the gradual addition of virtual machines to the virtualization pools. This pattern is not uncommon in DCs due to variations in the operational demands of server units, where power usage follows the dynamic nature of computational workloads.

### Identify potential EnPIs

The last step of the proposed methodology consists of identifying potential EnPIs for the studied DC room. A key in identifying potential EnPIs was investigating relevant EnPIs for DCs in scientific literature. In accordance with ISO 50,006^20^, EnPIs were developed for three levels: organizational, system and process. Additionally, energy performance parameters and their characteristics were used to establish the EnPIs. The analysis of the collected energy performance parameters was fundamental in developing EnPIs. The reason for this is because EnPIs in DCs are insufficiently explored in scientific literature, as well as the fact that the access of data describing EEU can vary between different DCs due to differences in measurement procedures. However, it should be noted as a limitation in our study that, as outlined in ISO 50,002^42^, improvements are typically implemented during the second step of an energy audit, whereas the analysis of EEU occurs in the first step, as in this research.

To develop and validate the proposed EnPIs, 11 workshops were held with consultants and employees from Ericsson between September 2020 and June 2023. Candidate EnPIs were proposed and discussed during the workshops and evaluated based on three main criteria: (i) relevance for representing key EEU processes, (ii) feasibility in terms of available operational data, and (iii) comparability across similar DC environments. The evaluation followed an iterative refinement approach, where indicators were continuously discussed, modified, and, where necessary, excluded. In cases of differing opinions, discussions were continued until agreement was reached among participants. It should be noted that only certain parts of these workshops focused on EnPIs; other topics discussed included energy system modeling and data collection, among others. These workshops played a crucial role in deepening the understanding of the collected data to tailor the EnPIs to the specific requirements of the studied DC room. Representatives from both academia and various segments of Ericsson, including facility management consultants, employees, and managers, attended all sessions to ensure a broad range of perspectives were incorporated into the discussions, facilitating comprehensive development of the EnPIs. The approach was inspired by the methodology employed by Andersson et al.^15^. Moreover, all participants in the workshops provided informed consent to participate and agreed that the information they shared could be used for research purposes. The discussions did not involve any sensitive personal data but focused on the participants’ actions and input in their professional roles and areas of expertise. All data have been anonymized, and the study was conducted in accordance with relevant guidelines. The experimental protocols were approved by the Technical Director at Tekniska Verken (Mile Elez), the Head of the Department of Management and Engineering at Linköping University (Louise Ödlund), and the Site Manager at Ericsson (Thomas Gotenstam).

**Fig. 3:**
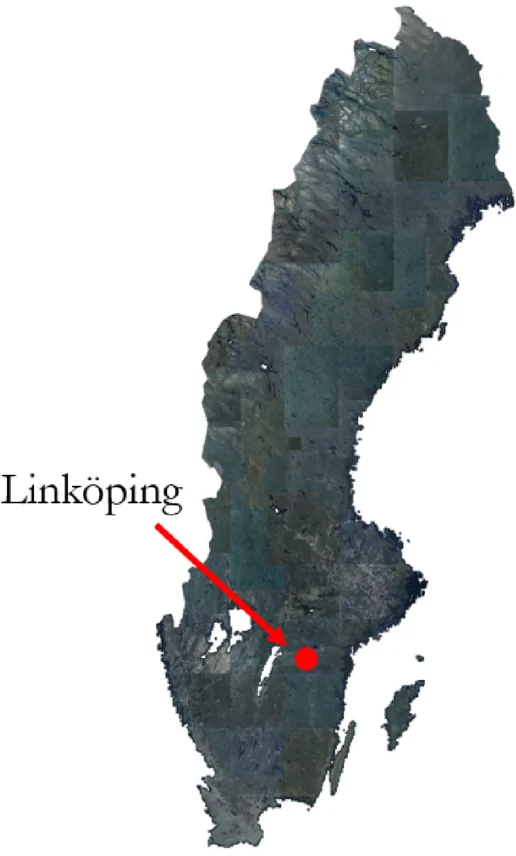
Location of Linköping in Sweden^42^.

The study object in this research is a DC room owned and operated by Ericsson in Linköping, Sweden. Figure 3 shows the location of Linköping within Sweden. Ericsson is a global telecommunications and technology company with extensive operations in information and communication technology. The selected DC room provides a real-world industrial case for investigating the energy use and operational characteristics of DC infrastructure.

**Fig. 4:**
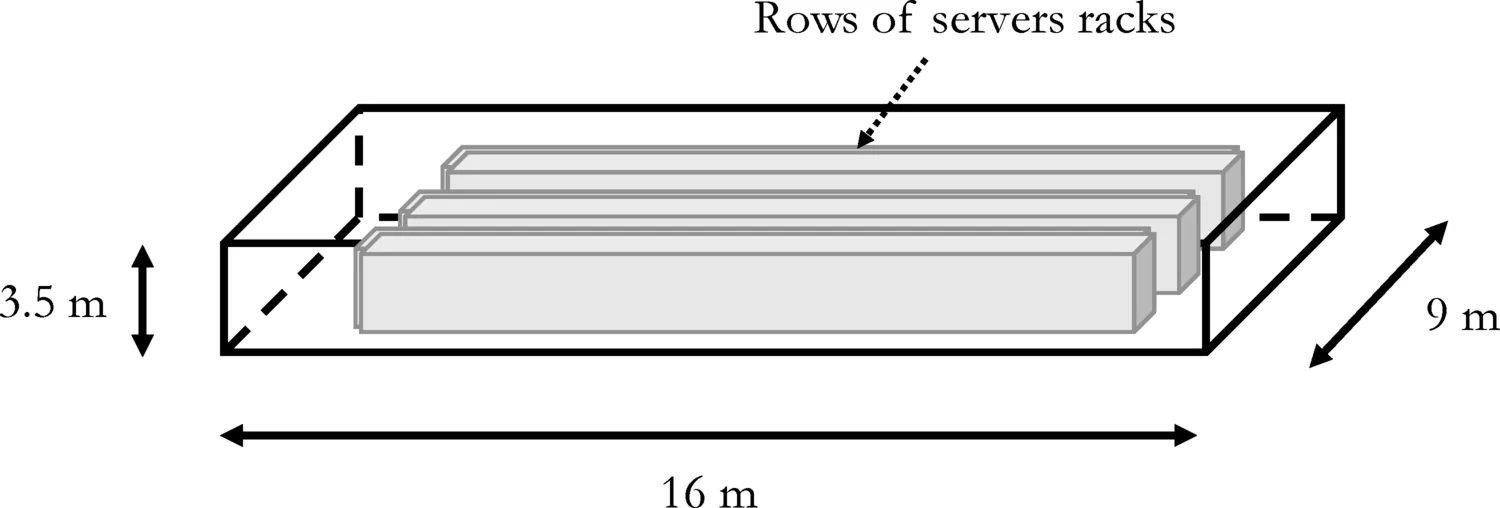
Visualization of the studied DC room.

Data of energy flows for the DC room has been gathered from 2021 to 01-01 to 2021–11-10. This corresponds to more than 90,000 data points with a time interval of five minutes. In total, 29 data measurement points were considered of which 27 described EEU processes, and two the total energy use for the DC room. Hence, more than 2.6 million data points were included in the development of the taxonomy. It is important to note that the analysis focuses on the classification of EEU processes and the identification of EnPIs within the studied DC room, rather than on seasonal variation in cooling performance. Since the DC room is located indoors and is surrounded by adjacent DC rooms, the room-level energy balance is not directly influenced by outdoor weather conditions. A visualization of the studied room for developing the taxonomy can be seen in Fig. 4, which has three rows of servers. Each row comprises seven Liquid Cooling Package (LCP) units resulting in a total of 21 LCPs in the room, which are cooled using district cooling. The room has an area of 144 m^2^ and a volume of 504 m^3^. Moreover, the rows of standard cabinets in the DC room measure 11.7 m in length, 800 mm in width, 1,200 mm in depth, and 2,000 mm in height. Each row contains 12 cabinets, except for one row, which contains 11 cabinets. Additionally, there are seven LCPs per row, distributed among the cabinets. Each cabinet holds between 20 and 40 standard off-the-shelf 1unit rack servers, along with network equipment.

## Results and discussion

### Taxonomy of EUU for a DC room

**Fig. 5:**
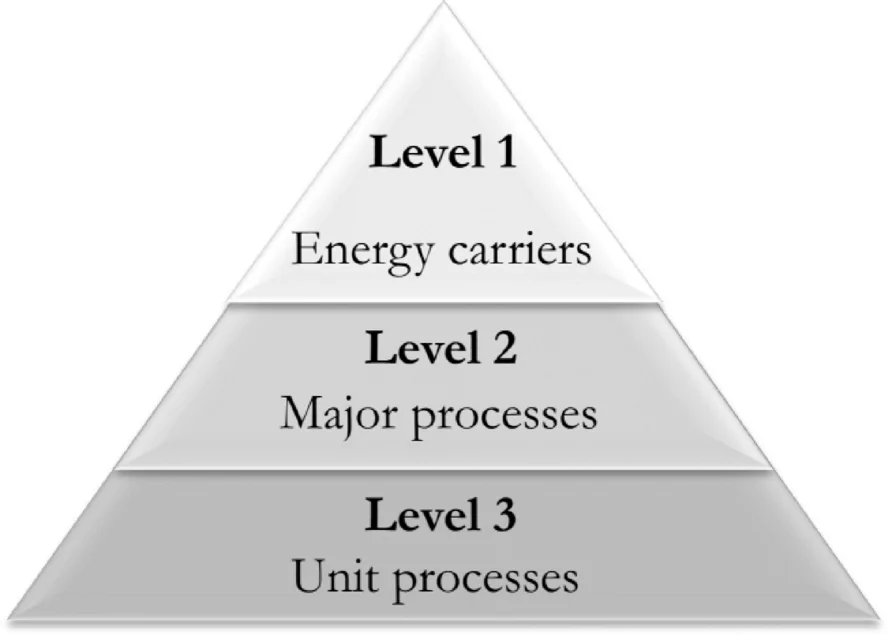
Hierarchical levels for energy use.

Inspired by Kanchiralla et al.^16^, and using energy flows as the attribute for a hierarchical architecture, has enabled differentiation of hierarchical levels for the studied DC. Following the analysis of energy flows as the attribute for classification, a three-level hierarchy has been framed, which can be seen in Fig. 5. The hierarchical structure provides a systematic representation of how energy flows are distributed from supply (energy carriers) to end-use processes. This enables identification of the main energy pathways within the DC room and supports a clear separation between data processing processes and support processes. Such structuring is important for analyzing energy performance in DCs, where energy use is often highly concentrated in a limited number of processes. Additionally, the proposed structure supports policy directions by enabling standardized energy reporting^44^.

Energy carriers, which correspond to the energy supply side, are Level 1 in the hierarchical classification. This level provides an outlook on how dependent the DC room is on various energy carriers, and the level only requires measurements at the energy supply point. In addition, as mentioned in “Development of taxonomy” Section, the accuracy of the EEU calculations can be assessed by comparing the aggregated energy use with the total energy use from all energy carriers. Level 2 corresponds to major processes which are data processing processes or support processes. Data processing processes are those processes that are used for value creation for Ericsson in the DC room, and support processes are linked with processes that provide support for the operation of the DC room. Moreover, the unit processes correspond to the Level 3 taxon, i.e., this level is connected to the objective of the various processes. Levels 2 and 3 have been determined based on Söderström^9^.

**Fig. 6:**
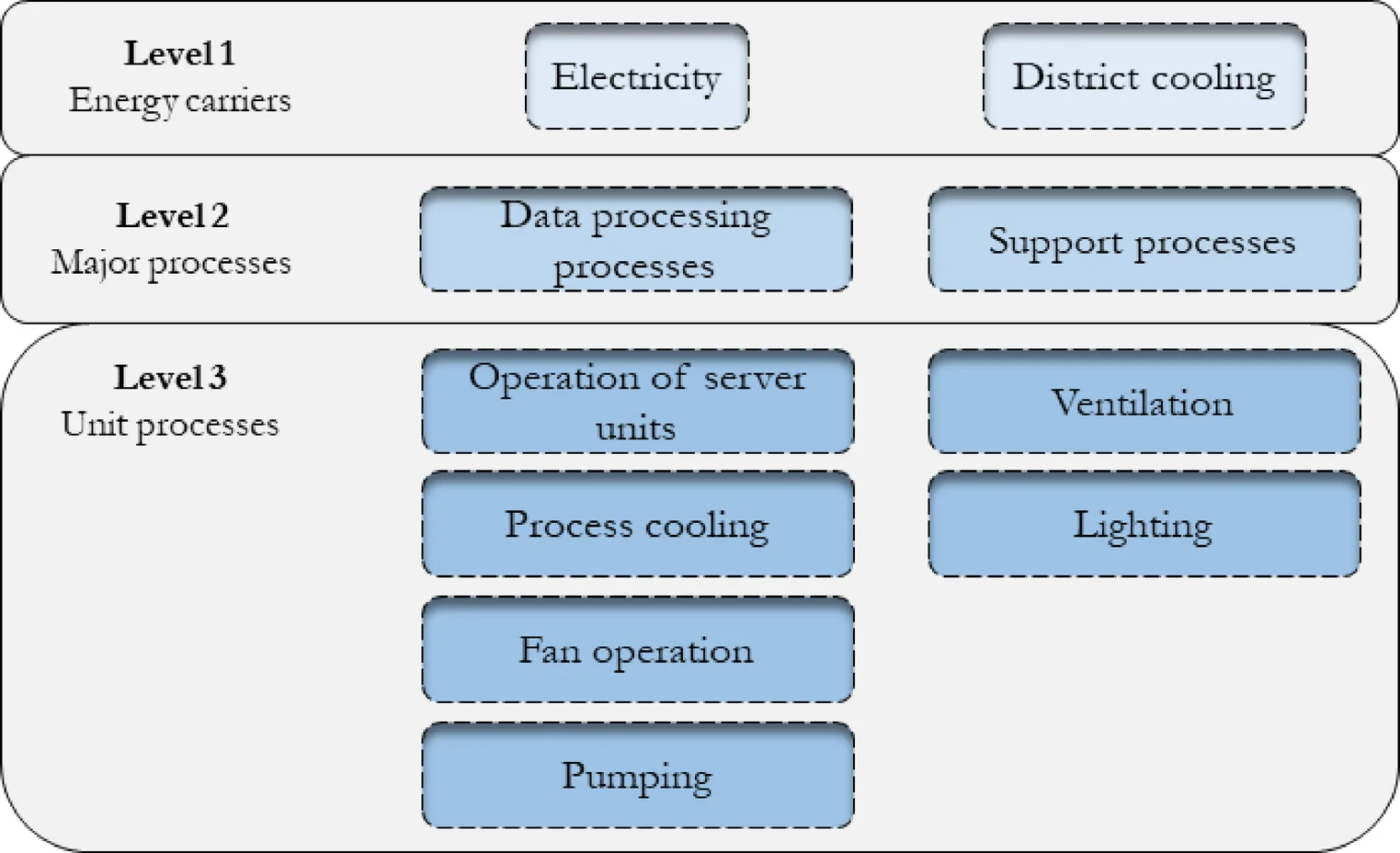
Schematic tree of the taxonomy for EEU in the studied DC room.

A novel taxonomy for EEU is developed for the studied DC room, as shown in Fig. 6. The schematic tree of the taxonomy has been developed by analysis of the collected energy flows, and it has been validated by Ericsson. Two energy carriers are included in Level 1, i.e., electricity and district cooling, representing the energy inputs to the system.

Level 2 is divided into either data processing processes or support processes, enabling a clear categorization of the DC room’s operational structure. At Level 3, four unit processes related to data processing processes are defined. These are operation of server units, process cooling, pumping and fan operation. Although process cooling, pumping and fan operation support the operation of the server units, they are classified as data processing processes in this DC-specific taxonomy because they are directly coupled to the operation and thermal management of the server equipment. In contrast, ventilation and lighting are classified as support processes since they provide general room-level services and are not directly linked to the data processing chain. Two processes connected to support functions are determined, which are ventilation and lighting. Each of these processes fulfills a specific role in the operation of the DC room.

The taxonomy provides a structured basis for distinguishing between major process categories and their associated unit processes in the DC room. This classification supports subsequent analysis of energy use and facilitates identification of the processes that should be prioritized in energy performance assessment and energy management. In addition, the taxonomy clarifies how the DC room can be differentiated into functionally distinct EEU processes, which is important for process-level monitoring and comparison. This provides a basis for interpreting later results on energy distribution and for establishing EnPIs at different system boundaries. It should be noted that sub-unit classification is not used because of the unavailability of data.

### EEU in the DC room

**Fig. 7:**
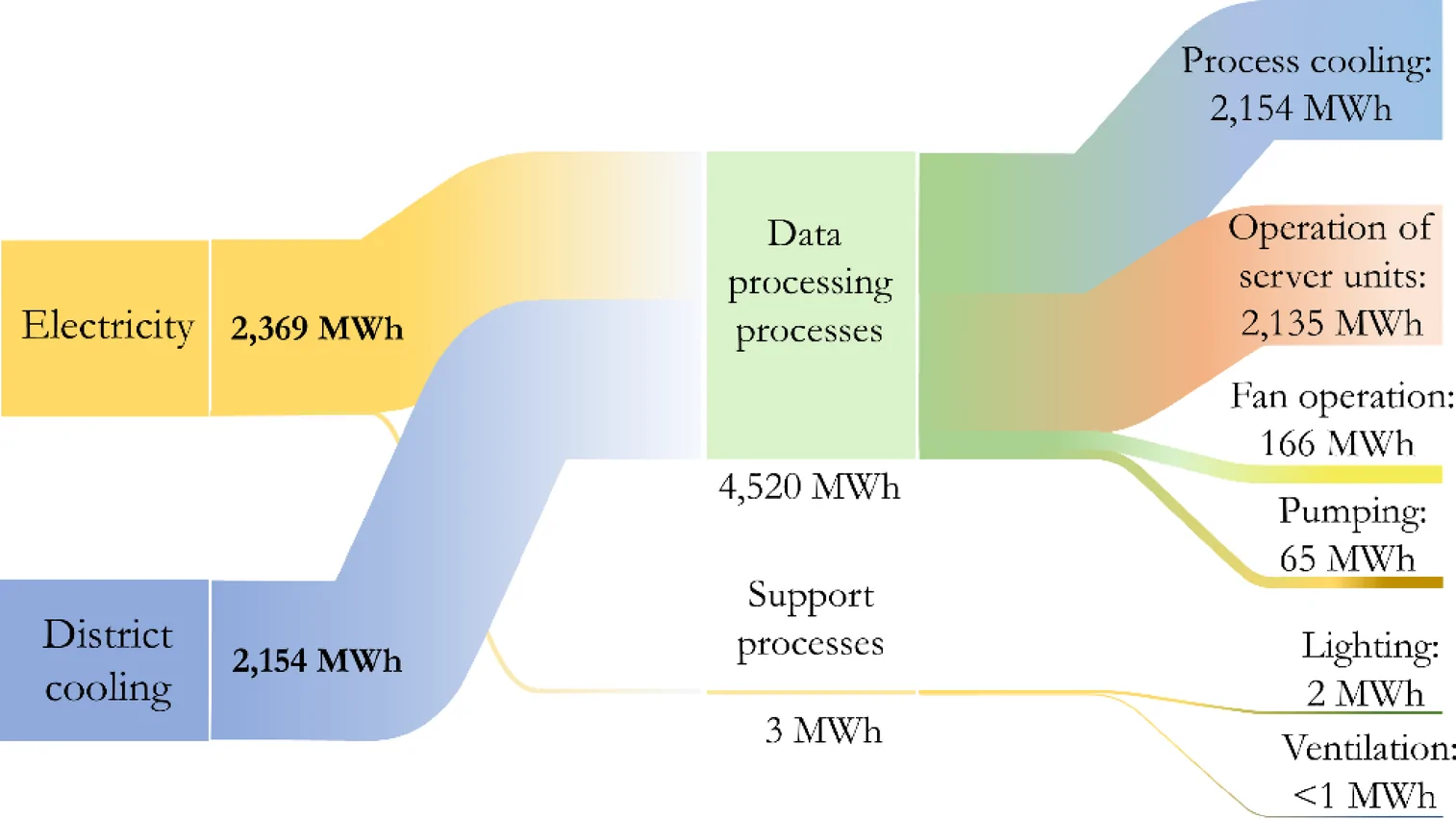
Energy flows for all levels in the hierarchical classification of the DC room.

Collected data of energy flows describing EEU are used to analyze energy use at different hierarchical levels according to Sect. 5.1. The annual energy flows in the DC room for all levels in the hierarchical classification can be seen in Fig. 7, as well as the distribution to various EEU processes. Total annual energy use is 4,523 MWh, of which 4,520 MWh and 3 MWh can be derived to data processing processes and support processes, respectively. Support processes are included in the taxonomy to provide a complete representation of the system boundary and to enable comparison with other DC configurations and industrial sectors. Their limited contribution to total EEU further clarifies that, in the studied water-cooled DC room, energy use is highly concentrated in processes directly related to data processing. It is important to highlight that this means that over 99.9% of the EEU is related to the data processing processes, which is a significantly higher figure compared to results reported in scientific literature on other industries. For example, production processes corresponding to 51% and 70% of total EEU have been reported for the Swedish engineering industry and the food industry^10,16^, and around 16% for the category “Others” in the pulp and paper industry, where “Other” includes both support process EEU as well as minor production process EEU^45^. The distribution of energy flows in this research clarifies the highly concentrated natures of energy use in DC environments, where a limited number of processes dominate total demand. In contrast to many industrial sectors, where support processes account for a more substantial share of energy use, the studied DC room is almost entirely driven by processes directly linked to data processing. This highlights the importance of focusing analytical and optimization efforts on these dominant processes when assessing energy performance. However, it is important to be aware of the fact that the research object consists of a single water-cooled DC room. Cooling strategies vary between different DCs and impact the distribution of EEU processes. Moreover, it can be noted that the EEU varies largely between the processes. The highest energy use is associated with process cooling (2,154 MWh), followed by electricity usage for server unit operation (2,135 MWh), while the lowest energy use is for ventilation (0.9 MWh). This significant difference is due to the substantial cooling demand of servers and the relatively low ventilation requirements in the DC room.

**Fig. 8:**
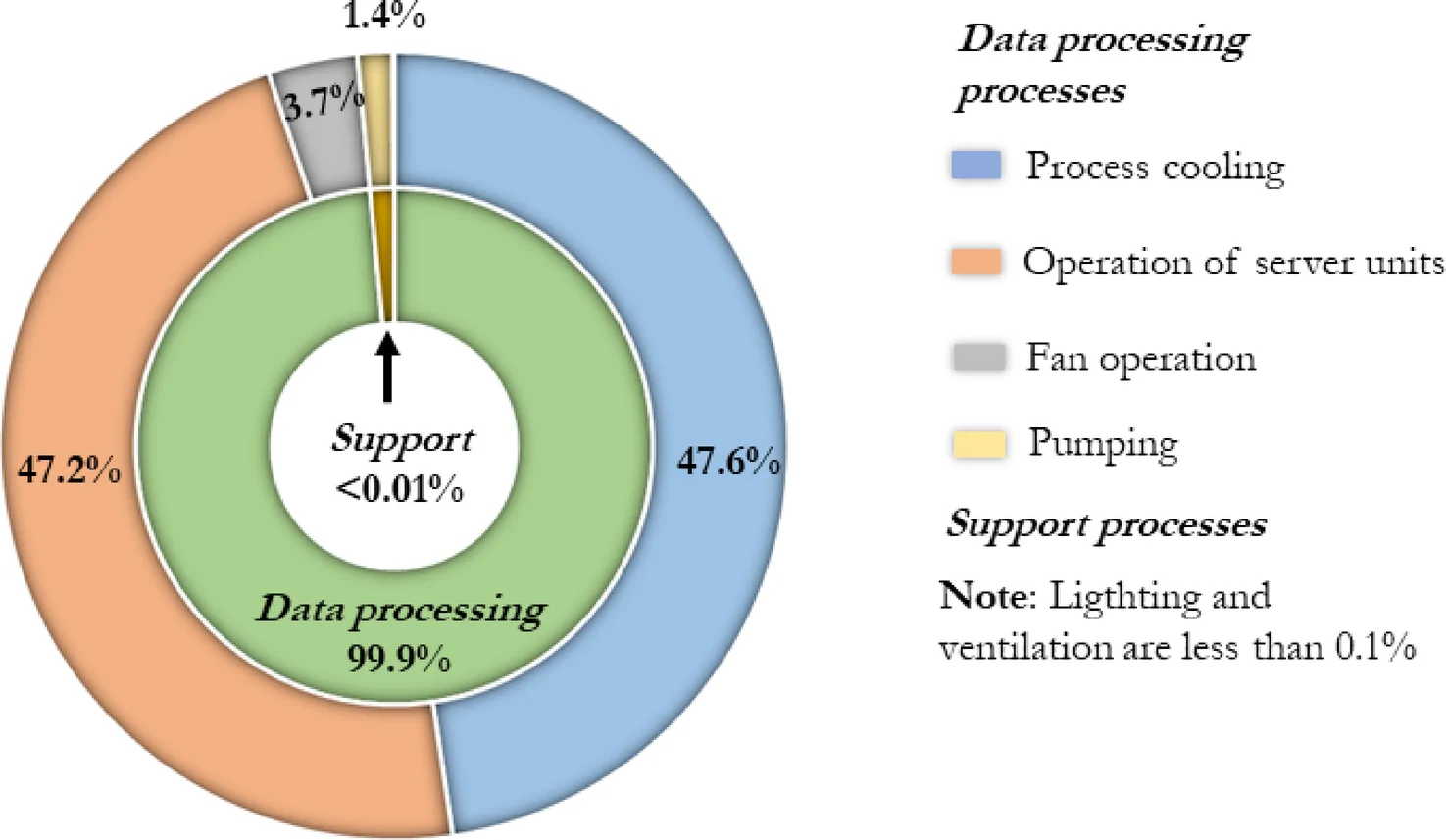
Percentage share of EEU for various data processing and support processes in the DC room. It can be noted that lighting and ventilation correspond to less than 0.1% of the energy use.

The percentage share of EEU for the data processing and support processes at Level 3 can be seen in Fig. 8. Process cooling and operation of server units are the most energy-intensive processes corresponding to 95% of total EEU in the DC room. The reason for this is the high energy use needed for supply of electricity and cooling to support a proper and continuous operation of server equipment. This strong concentration shows a clear coupling between computational demand and cooling requirements, where increases in server activity directly translate into increased cooling demand. The relatively small contribution from other processes further emphasizes that improvements in overall energy performance are largely dependent on optimizing these two processes. This also suggests that detailed monitoring and control of cooling behaviour is important, as even minor energy efficiency gains in these dominant processes can result in significant reductions in total energy use. Moreover, the least energy-intensive processes are connected to the support processes, i.e., lighting and ventilation, which correspond to less than 0.1% of total EEU. In this research, the EEU for lighting is approximated using the power utility of the bulbs and the operational hours (1,500 h). These figures are in accordance with approximations for guidelines of installed power for lighting in industry^46^. Similarly, the electricity used for the ventilation is calculated using data about the specific fan power, which is a parameter describing the energy-efficiency of ventilation systems, and the total ventilation flow. The EEU for pumping is derived from the share of water flow supplied to the studied DC room from the circulation system. With regards to fan operation, the EEU is calculated using the electricity use for the entire DC room deducted by the electricity for server operation and lighting. The reason for the approximations of the abovementioned EEU process and not using collected EEU data is due to practical constraints. However, since the EEU for ventilation, lighting, pumping and fan operations correspond to solely 5% of total EEU and the approximations are generally based on site-specific information, such as actual data about installed power for lighting, the approximations of EEU for the unit processes allow for a good representation of the actual EEU. These results contribute beyond existing studies, which primarily focus on system- or component-level classifications^47–49^, by introducing a process-oriented taxonomy that explicitly links EEU processes to hierarchical levels and supports their direct operationalization through EnPIs.

The limitations of these results are that they are based on one DC room with a specific cooling strategy at a single DC site, and are also constrained by limited detailed measurement capabilities. Consequently, the findings are not fully representative of the entire DC sector. The generalizability of taxonomies in various sectors is problematic in its nature. This is because of large differences in mainly production processes from one industrial sector to another. Therefore, there is a need to develop industry-specific taxonomies, which is especially the case for DCs considering the unique operational and production characteristics compared to other industries. This research, taking into consideration its limitations in terms of generalizability, is one of the first attempts to provide such a taxonomy for the DC sector, and the analysis is helpful in the guidance of establishing a future common hierarchical architecture for DCs. Moreover, in this study, all major EEU processes are powered by electricity, except for district cooling. This must be taken into account when analyzing the findings beyond the studied DC and the Swedish context. It should also be noted that the analysis is based on operational data from 2021. While DC technologies and operating conditions have evolved, the fundamental structure of energy use across EEU processes remains consistent. The identified distribution of energy use, dominated by server operation and process cooling, reflects underlying system requirements that are common across DC environments. Although the absolute values of the EnPIs may change over time due to increasing computational demand and higher power densities, this does not affect the relative importance of the main EEU processes. Moreover, future advances in cooling, fan, and pumping system efficiency may influence the relative distribution of EEU over time. Nonetheless, the results indicate that the main EEU processes remain dominant under the studied conditions. The results are therefore considered representative in terms of process structure, even if the magnitude of energy use evolves over time.

### EnPIs in the DC room

To monitor the energy efficiency of the water-cooled DC room, a number of EnPIs are suggested that are provided in Tables 2, 3 and 4 grounded in the workshops outlined previously in “Identify potential EnPIs” Section. These are differentiated depending on system boundary in line with ISO 50,006^20^. The EnPIs are established to allow for a characterization of the various EEU processes, as well as to describe the overall energy performance of the DC room. This has been achieved by using collected data on energy flows, investigating EnPIs from other sectors, and by analyzing common performance indicators used in DCs. The identified EnPIs are crucial in monitoring and identifying hotspots in the EEU of the support and focus processes, primarily the most energy-intensive processes, i.e., the operation of server units and process cooling which correspond to 95% of total energy use as mentioned in the previous section. All developed EnPIs were validated by facility management consultants at the site and operation managers from the case company Ericsson. Ericsson expresses a higher interest in EnPIs related to process cooling, due to fluctuations in cooling behaviour of the LCP units and because process cooling almost corresponds to half of the total EEU. This indicator could also be used to compare or choose different process cooling strategies. The proposed EnPIs are based on commonly available operational data, including energy use, power demand, and system-specific parameters, and are intended for applications such as performance monitoring, benchmarking, and identification of energy-intensive processes. The proposed EnPIs are defined based on the structure of the EEU processes and the operational parameters required to monitor them, rather than on the absolute energy values from 2021. Therefore, the EnPI framework remains applicable to DC rooms with comparable infrastructure and data availability, while the numerical results should be interpreted as case-specific for the investigated period. Moreover, a time-resolved data resolution, for example on the order of 5 min, is recommended to adequately capture variations in system operation, although the appropriate temporal resolution depends on the dynamic characteristics of the DC.

**Table 2:** Suggested EnPIs to monitor on an organizational level.

Organizational level	
*EnPI*	*Unit*
Total specific energy use	kWh/(m^2^∙year)
Specific energy use for cooling	kWh/(m^2^∙year)
Specific energy use for electricity	kWh/(m^2^∙year)
Total peak power demand	kW
Peak power demand for cooling	kW
Peak demand for electricity	kW
PUE	(-)

**Table 3:** Suggested EnPIs to monitor on a system level.

System level	
*EnPI*	*Unit*
Electricity use for server operation	kWh
Energy use for process cooling	kWh
Ratio between cooling and electricity use (Energy_cooling_/Energy_electricity_)	(%)
Peak power demand for server operation	kW
Peak cooling demand for process cooling	kW
Performance per watt	Flops/W

**Table 4:** Suggested EnPIs to monitor on a data processing process and support process level.

Data processing and support level		
*Data processing process level*	*EnPI*	*Unit*
	Energy use for process cooling	kWh
	Peak cooling demand for process cooling	kW
	Power for pumping	kW
*Support process level*	*EnPI*	*Unit*
	Power	(kW)
	Flow rate for ventilation	(m^3^/s)
	Specific energy use for lighting	kWh/(m^2^∙year)

EnPIs on an organization level are established with basis on the specific energy use and peak power demand for cooling and electricity of the DC room, and the PUE value. Consequently, an overall picture of the DC room’s energy performance can be obtained. The reason for developing EnPIs per m^2^ is partly that area-based indicators are commonly used in other industries and can support comparisons of space-related energy intensity. However, DCs, such indicators should be interpreted together with DC-specific EnPIs related to IT load, cooling demand, and operational performance. With regard to EnPIs per m^2^ in DCs, these indicators are of specific interest if there is a lack of facility area. The suggested EnPIs on a system level are similarly developed based on data for electricity and cooling but delimited to the server rows in the DC room. However, as can be seen in Table 3 the performance per watt, Flops/W, is also included as an EnPI on a system level. This metric describes the energy performance for a specific computer architecture, or cluster, that can solely be determined for the set cluster nodes in the DC room. At the second level, the EEU processes are differentiated into data processing and support processes. At the third level, these are further divided into available unit processes, such as server operation, process cooling, ventilation, lighting, pumping, and fan operation. However, EnPIs for individual server units are not available due to limitations in the measurement of server-level electricity use. Moreover, these results contribute beyond existing studies, which primarily focus on system- or component-level classifications^47–49^, by introducing a process-oriented taxonomy that explicitly links EEU processes to hierarchical levels and supports their direct operationalization through EnPIs. This also aligns with recent EU assessments of DC energy performance, which emphasize the importance of standardized metrics and improved comparability across DC environments^50^.

The proposed taxonomy and EnPI framework serve different but complementary purposes: the taxonomy classifies EEU into structured process levels, while the EnPIs quantify energy performance at defined monitoring boundaries. The taxonomy provides a structural classification of EEU, from energy carriers (Level 1) to major process categories (Level 2), including both data processing and support processes, and further to unit processes (Level 3), whereas the EnPIs define performance at different monitoring boundaries. Organizational-level EnPIs span all taxonomy levels and reflect the performance of the entire DC room. System-level EnPIs are defined for subsystems within the main process categories (Level 2) and are derived from one or more underlying unit processes (Level 3). In the present study, these are primarily associated with data processing processes due to their dominant contribution to total energy use. Some indicators, such as the ratio between cooling and electricity use, combine energy from multiple unit processes, for example server operation and process cooling, while others, such as Flops/W, are linked to server operation but evaluated at a system level. Process-level EnPIs correspond directly to individual unit processes (Level 3), including process cooling, pumping, ventilation, and lighting, enabling more detailed analysis of specific EEU processes.

### Policy implications

Taxonomies allow for outlining energy use from different sectors, and for valid comparison between different industries^10^. Hence, decision-makers and legislators can use categorization of EEU data to identify processes in various sectors with the highest energy efficiency potential. To the best of the authors’ knowledge, this research represents one of the initial efforts to design a taxonomy for DCs that includes EEU related to both cooling and electricity. Moreover, EnPIs are a crucial part of successful energy management^51^. By identifying the processes with the highest energy use in a DC facility, policymakers can use this information to support targeted energy-efficiency measures. In addition, server units and cooling units that have a malfunctioning operating system can be identified and further analyzed to increase the overall energy efficiency of the DC room or facility. The development of a taxonomy in DCs facilitates benchmarking of relevant processes and can be used as support in decision-making for improving energy performance. The lack of data supporting benchmarking between EEU processes in industries has previously been pointed out by May et al.^52^. In addition, a common framework for classification of economic activities can be of great importance to implement the European Green Deal^7^, and to allow for effective communication of DC performance.

The schematic tree presented in Fig. 5 can be used as inspiration for categorization and comparison of EEU processes in water-cooled DC rooms, as well as a systematic approach for differentiating energy flows. This means that the developed taxonomy is transferable to DC rooms with similar cooling techniques. However, the proposed structure is intended to be adaptable to different DC room classes. Depending on the cooling configuration, the unit processes and corresponding EnPIs can be adjusted to include, for example air-cooled systems. In addition, energy carriers for cooling differ between DCs due to local variations in climate and fuel prices. Nonetheless, this research provides a contribution for classification of EEU in DCs, as well as an insight into differences of EEU related to data processing and support processes in the DC sector compared to other industries. This stresses the need for developing and applying customized energy management strategies for different sectors. In addition, the creation of an architecture database describing EEU supports the monitoring of processes.

EnPIs at different levels, i.e., system, organization, and process level, provide analysis of the energy performance of a DC from different perspectives based on set system boundaries. Schulze et al.^53^ state in their review of energy management in industry that EnPIs are commonly more available on an aggregated level, overlooking EnPIs on sublevels. This is despite that sublevel EnPIs are helpful in pinpointing significant energy efficiency opportunities^54^. On an organizational level, an overall outlook on the energy performance of the organization, in this case the water-cooled DC room, can be provided. This is useful for tracking the energy performance of the organization, as well as to use for benchmarking between different DC rooms or facilities. Hence, both external and internal benchmarking is made possible. External benchmarking can be used for e.g., comparison with best practices within the industry, and internal benchmarking is valuable for identifying DC rooms which are objects for energy efficiency within the company. Consequently, both external and internal benchmarking on an organizational level can assist in reducing expenditures related to energy use, as well as fulfill regulations regarding environmental performance and energy use. By setting the system boundary around interacting processes such as cooling systems, EnPIs on a system level can be determined. System level EnPIs allow for identifying and targeting specific systems for upgrade and energy efficiency. By setting the system boundary around specific processes in DC room, such as LCP units, EnPIs on a process level can be generated. Based on collected EEU data, process level EnPIs allow for identifying specific processes with major energy savings potential. However, in practice, process level EnPIs frequently entail significant effort to collect adequate and relevant data on energy use^54^.

## Conclusion

To increase the awareness of energy characteristics in DCs and improve energy management, categorization of EEU processes in the DC sector can assist in increasing the awareness of the most energy-intensive processes and improving energy management. The research contributions of this study are twofold: firstly, a novel adaptation of hierarchical taxonomies for DCs with detached in-rack water cooling systems; and secondly, to identify the most relevant EnPIs. Moreover, this research offers new insight into the research field of taxonomies by the classification of energy use related to both cooling and electricity in the DC sector.

The developed hierarchical taxonomy and categorization of EEU processes clarifies the most energy-intensive processes in the DC room. The taxonomy consists of four data processing processes, i.e., operation of server units, process cooling, pumping and fan operation, and two support processes, i.e., lighting and ventilation. More than 99.9%, 4,520 MWh of total energy use is related to data processing processes. Process cooling and the operation of server units together account for 95% of the total EEU, with process cooling at 2,154 MWh and server unit operation at 2,135 MWh. Consequently, energy management strategies for DC rooms should focus on EEU processes that are both energy-relevant and operationally actionable. In the studied case, process cooling and operation of server units are identified as central processes, while the relevance of specific processes may vary depending on the DC room infrastructure, cooling configuration, and available measurement data. In addition, the large discrepancy on the distribution between production, in this research labeled data processing, and support processes in the studied DC room compared to other industries sheds light on the need for an industry-specific taxonomy in the DC sector. The suggested EnPIs at different levels allow for analysis of performance metrics of a DC from different perspectives. This is useful for both external and internal benchmarking with DCs that have similar cooling strategies. Monitoring energy use of various processes is another key benefit using the established EnPIs. Other potentialities in generating EnPIs include identifying individual equipment that are target for further analysis and energy efficiency.

The present study contributes to the existing knowledge base in industry by providing a unique taxonomy for DCs and categorization of EEU processes connected to cooling and electricity, based on a methodology that has been applied in other types of industries. The study demonstrates that this approach can be successfully adapted and applied to DCs, offering a novel way to analyze and categorize EEU processes in this specific context. In addition, this research can be used as inspiration for analysis of EEU processes in other DCs. The approach used is replicable for investigating other DCs, also with different cooling techniques. However, since the study is based on a DC room with a detached in-rack water cooling system, the results are primarily relevant to DCs using similar cooling systems. While the method remains adaptable, the findings are most applicable to DCs with comparable infrastructure. Access to measurement data for EEU processes is also a critical prerequisite for conducting this kind of analysis.

Lastly, it is important to acknowledge the limitations of this study. The analysis is based on a DC room and operational data from 2021, which may limit generalizability to other configurations and operating conditions. In addition, some EEU processes were estimated indirectly due to measurement constraints. While these limitations introduce some uncertainty, they do not affect the overall applicability of the proposed methodology. Moreover, future research should focus on applying and validating the framework across multiple DCs with varying configurations, integrating more detailed measurements, and extending the analysis toward subsequent stages of the energy audit, where the implementation of improvement measures are typically addressed.

## Data Availability

The data that support the findings of this study belong to the company Ericsson, but restrictions apply to the availability of these data, which were used under license for the current study, and are therefore not publicly available. However, the data are available from the main author V. Milić upon reasonable request and with permission from the company Ericsson.
